# Supplementary material for: Career choices of underrepresented and female postdocs in the biomedical sciences
Source: eLife. 2020 Jan 3;9:e48774. doi: 10.7554/eLife.48774 (PMC6977964; doi:10.7554/eLife.48774)
Supplement: Supplementary file 1. [file elife-48774-supp1.pdf]

U-MARC: Understanding Motivations for Academic Research Careers

Informed Consent

TITLE OF STUDY

U-MARC Study: Understanding Motivations for Academic Research Careers

PRINCIPAL INVESTIGATOR

Linnie M. Golightly, M.D. Associate Dean for Diversity Associate Professor of Clinical Medicine, Microbiology & Immunology Weill Cornell Medicine 1300 York Avenue, Room A-421 New York, New York 10065

W. Marcus Lambert, Ph.D. (co-PI), Director of Diversity and Student Services Weill Cornell Graduate School of Medical Sciences Weill Cornell Medicine 1300 York Avenue, Room A-131 New York, New York 10065

PURPOSE OF STUDY

You are being asked to take part in a research study. Before you decide to participate in this study, it is important that you understand why the research is being done and what it will involve. Please read the following information carefully. Please ask the researcher if there is anything that is not clear or if you need more information.

Dr. W. Marcus Lambert (Weill Cornell Medicine) and Dr. Linnie Golightly (Weill Cornell Medicine) are conducting a study to better understand the factors that influence interest in an academic research career. You are being asked to participate in this research study because you are conducting a biomedical or biological postdoctoral fellowship at an academic institution.

NUMBER OF SUBJECTS

We project a sample size of 300-350 participants for this study.

STUDY PROCEDURES

If you agree to participate, you will be asked to complete a survey that includes questions about your career intention, your postdoc experience, and factors that have influenced your career choice. The survey will take approximately 10-15 minutes to complete.

This survey is anonymous and no identifying information will be associated with your survey responses.

RISKS

Participants in this study may perceive some questions as sensitive.

You may decline to answer any or all questions and you may terminate your involvement at any time if you choose.

BENEFITS

There will be no direct benefit to you for your participation in this study. However, we hope that the information obtained from this study will bring benefit to others as they make more informed choices about the career options available to them after a PhD.

CONFIDENTIALITY

Your responses to this survey will be anonymous. Please do not write any identifying information on your survey. This signed Consent and the Contact Information post-survey are submitted separately from the U-MARC Survey so that we will not identify survey responses.

COMPENSATION

To thank you for participating in the survey, we will be awarding three $100 Amazon e-gift cards via a random drawing from all respondents who complete the post-survey contact information form.

You will not be eligible for compensation if you do not complete the survey and the post-form so that we know where to send your gift card if you win the drawing. This U-MARC survey is anonymous and no identifying information will be associated with your survey responses. The drawing will take place after the closer of the survey.

CONTACT INFORMATION

If you have questions at any time about this study, or you experience adverse effects as the result of participating in this study, you may contact the researcher whose contact information is provided on the first page. If you have questions regarding your rights as a research participant, or if problems arise which you do not feel you can discuss with the Primary Investigator, please contact the Institutional Review Board at (865) 354-3000, ext. 4822.

VOLUNTARY PARTICIPATION

Your participation in this study is voluntary. It is up to you to decide whether or not to take part in this study. After you sign the consent form, you are still free to withdraw at any time and without giving a reason. Withdrawing from this study will not affect the relationship you have, if any, with the researcher.

CONSENT

I have read and I understand the provided information and have had the opportunity to ask questions. I understand that my participation is voluntary and that I am free to withdraw at any time, without giving a reason and without cost. I understand that I will be given a copy of this consent form. I voluntarily agree to take part in this study.

Add signature to proceed

____

Education and Experience

Highest degree earned:

- ☐ PhD
- ☐ MD (not PhD)
- ☐ MD/PhD
- ☐ Other

Are you a 'postdoc' (Postdoctoral Researcher/Fellow)?

- ◯ Yes
- ◯ No, I have another classification/title

Please indicate your classification/title (e.g., Research Associate) ______________________________

Please indicate the date you started your PhD training. ______________________________
((To the best of your knowledge.))

Please indicate the date you defended your PhD. ______________________________
((To the best of your knowledge.))

Time to degree ______________________________

Time post-PhD ______________________________

Please indicate your current academic institution. ______________________________

Please indicate the date you started your current (postdoctoral) position. ______________________________
((To the best of your knowledge.))

Current postdoc length ______________________________

How many postdoctoral positions have you held (including your current position, if your current position is a "postdoc")? ______________________________

Please select your primary (or best fitting) field of study:

◯ Anatomy
◯ Bacteriology
◯ Biochemistry
◯ Bioinformatics
◯ Biology/Biomedical Sciences, General
◯ Biology/Biomedical Sciences, Other
◯ Biomedical Sciences
◯ Biometrics & Biostatistics
◯ Biophysics
◯ Biotechnology
◯ Botany/Plant Biology
◯ Cancer Biology
◯ Cell/Cellular Biology & Histology
◯ Computational Biology
◯ Developmental Biology/Embryology
◯ Ecology
◯ Endocrinology
◯ Entomology
◯ Environmental Toxicology
◯ Epidemiology
◯ Evolutionary Biology
◯ Genetics/Genomics, Human & Animal
◯ Immunology
◯ Marine Biology & Biological Oceanography
◯ Microbiology
◯ Molecular Biology
◯ Neurosciences & Neurobiology
◯ Nutrition Sciences
◯ Parasitology
◯ Pathology
◯ Pharmacology
◯ Physiology
◯ Plant Genetics
◯ Plant Pathology/Phytopathology
◯ Plant Physiology
◯ Structural Biology
◯ Toxicology
◯ Virology
◯ Wildlife Biology
◯ Zoology
◯ Other

How many publications do you have (published and under review)?

(Place a mark on the scale above)

Please indicate your number of first author publications.

(Place a mark on the scale above)

First author publication rate (# of first author publications/years in research): ________________________________

Of the journals in which you have published, what is the highest impact factor? (Google: name of journal + "impact factor") ________________________________
((i.e., For PNAS, enter: 9.4))

Demographic Information - (Confidentiality is important to us. Your identify will remain anonymous.)

Are you a U.S. citizen / permanent resident of the United States?
- ◯ Yes
- ◯ No

Ethnicity:
- ◯ Hispanic or Latino
- ◯ Not Hispanic or Latino

Race:
- ☐ American Indian or Alaskan Native
- ☐ Asian
- ☐ Black or African American
- ☐ Native Hawaiian or Other Pacific Islander
- ☐ White

To which gender identity do you most identify?
- ◯ Female
- ◯ Male
- ◯ Other

If "other", please specify: ________________________________

How do you self-identify?
- ◯ Straight or Heterosexual
- ◯ Gay or Lesbian
- ◯ Bisexual
- ◯ Other

If "other", please specify: ________________________________

Marital status:
- ◯ Single
- ◯ Married
- ◯ Domestic Partner/Living with Partner

Do you have children or dependents?
- ◯ Yes
- ◯ No

Have you ever received or been eligible for a low-income (need-based) grant or loan as a student?
- ◯ Yes
- ◯ No

Select the highest grade level completed by your parents.
- ◯ Did Not Complete High School
- ◯ High School/GED
- ◯ Some College
- ◯ Associate's Degree
- ◯ Bachelor's Degree
- ◯ Master's Degree
- ◯ Doctorate

How would you rate your understanding of the careers available to you?
- ◯ Significant
- ◯ Moderate
- ◯ Very little

Please rank the following career paths from Most likely to pursue to Least likely to pursue. (Only one selection is allowed per column.)

	Most likely to pursue	More likely to pursue	Equally likely to pursue	Less likely to pursue	Least likely to pursue
Academic (Faculty), Research-Intensive	○	○	○	○	○
Academic (Faculty), Teaching-Intensive	○	○	○	○	○
Other Research-Intensive (e.g. Industry)	○	○	○	○	○
Non-Research, Science-Related	○	○	○	○	○
Non-Science Related	○	○	○	○	○

How has your commitment to this career path changed, since starting your first postdoc?

	More committed	Stayed the same	Less committed
Academic (Faculty), Research-Intensive	○	○	○
Academic (Faculty), Teaching-Intensive	○	○	○
Other Research-Intensive (e.g. Industry)	○	○	○
Non-Research, Science-Related	○	○	○
Non-Science Related	○	○	○

Please rate how strongly you disagree or agree with the following statements:

	Strongly Disagree	Disagree	Neutral	Agree	Strongly Agree
My career choice is highly influenced by the current grant funding climate.	◯	◯	◯	◯	◯
Guidance from my lab PI has highly influenced which career path I will pursue.	◯	◯	◯	◯	◯
Guidance from mentors other than my PI has highly influenced which career path I will pursue.	◯	◯	◯	◯	◯
My career choice is highly influenced by my passion for science and discovery.	◯	◯	◯	◯	◯
My career choice is highly influenced by my success thus far in research (grants, publications, awards).	◯	◯	◯	◯	◯
My career choice is highly influenced by my personal life circumstances.	◯	◯	◯	◯	◯
My career choice is highly influenced by the job prospects my field.	◯	◯	◯	◯	◯
My career choice is highly influenced by my immigration status or citizenship.	◯	◯	◯	◯	◯

You mentioned that your career choice is highly influenced by your personal life circumstances. Please explain. ______________________________

Influential factors Please rate how strongly you disagree or agree with the following statements:

	Strongly Disagree	Disagree	Neutral	Agree	Strongly Agree
Financial security is one of the top reasons for my career choice.	○	○	○	○	○
My career choice is highly influenced by the career choices of my friends.	○	○	○	○	○
My career choice is highly influenced by my responsibility to my family (significant other/spouce, children, and/or other dependents).	○	○	○	○	○
My career choice is highly influenced by the lifestyle of those in the positions that I want.	○	○	○	○	○
My career choice is highly influenced by the impact that I can make on society or to a community.	○	○	○	○	○
My career choice is highly influenced by the prestige that comes with the field.	○	○	○	○	○
My career choice is highly influenced by my racial/ethnic representation in science.	○	○	○	○	○

Outcome expectations Please rate how strongly you disagree or agree with the following statements:

	Strongly Disagree	Disagree	Neutral	Agree	Strongly Agree
If I work hard in the lab I can expect a publication in a top journal.	○	○	○	○	○
There is a good correlation between my work effort and performance in the lab.	○	○	○	○	○
I will be successful in receiving grants and funding as long as I put in the right amount of effort.	○	○	○	○	○
Publications in top journals will lead to a faculty position.	○	○	○	○	○
Independent grant funding will lead to a faculty position.	○	○	○	○	○
A research-intensive faculty position would require more than one postdoc position.	○	○	○	○	○
A research-intensive faculty career would be very satisfying for me.	○	○	○	○	○
A research-intensive faculty career would increase my sense of self-worth.	○	○	○	○	○
My peers would think highly of me if I obtain a research-intensive faculty position.	○	○	○	○	○
Pursuing a research-intensive faculty position would enable me to associate with the kind of people that I value most.	○	○	○	○	○

Research self-efficacy Please rate how strongly you disagree or agree with the following statements:

	Strongly Disagree	Disagree	Neutral	Agree	Strongly Agree
I have the ability to have a successful career as a researcher.	○	○	○	○	○
I can publish in top scientific journals like Science, Nature, and Cell.	○	○	○	○	○
I am confident that I can secure grants in my field.	○	○	○	○	○
I can develop novel and successful research ideas.	○	○	○	○	○
I can mentor and supervise others in their training.	○	○	○	○	○

What advice would you give to someone thinking about an academic research career?

____

Finally, we are considering mechanisms to better support academic career interests. Would any of the following increase your likelihood to pursue an academic research career? Please rate how strongly you disagree or agree with the following items.

	Strongly Disagree	Disagree	Neutral	Agree	Strongly Agree
A course on transitioning to research independence that covers all aspects of obtaining a faculty position would increase my likelihood to pursue an academic research career.	○	○	○	○	○
A course on how to teach science and the pedogy of scientific teaching would increase my likelihood to pursue an academic research career.	○	○	○	○	○
A defined mentor outside of my lab that would help in the step-by-step guidance of my career would increase my likelihood to pursue an academic research career.	○	○	○	○	○
Training in and opportunities to conduct health disparities research would increase my likelihood to pursue an academic research career.	○	○	○	○	○
Specific educational training that would permit the application of basic science principles in community-based settings would increase my likelihood to pursue an academic research career.	○	○	○	○	○
Fellowships and grants to support URM investigators would increase my likelihood to pursue an academic research career.	○	○	○	○	○
